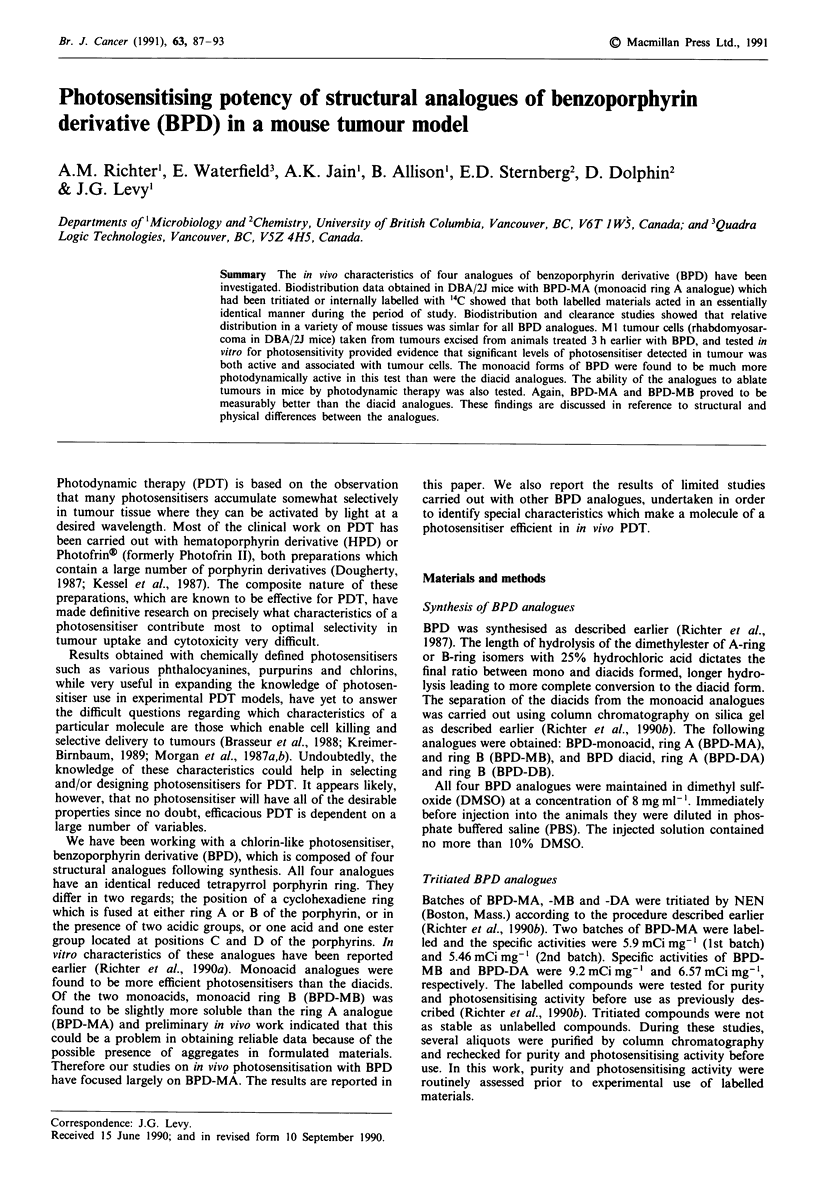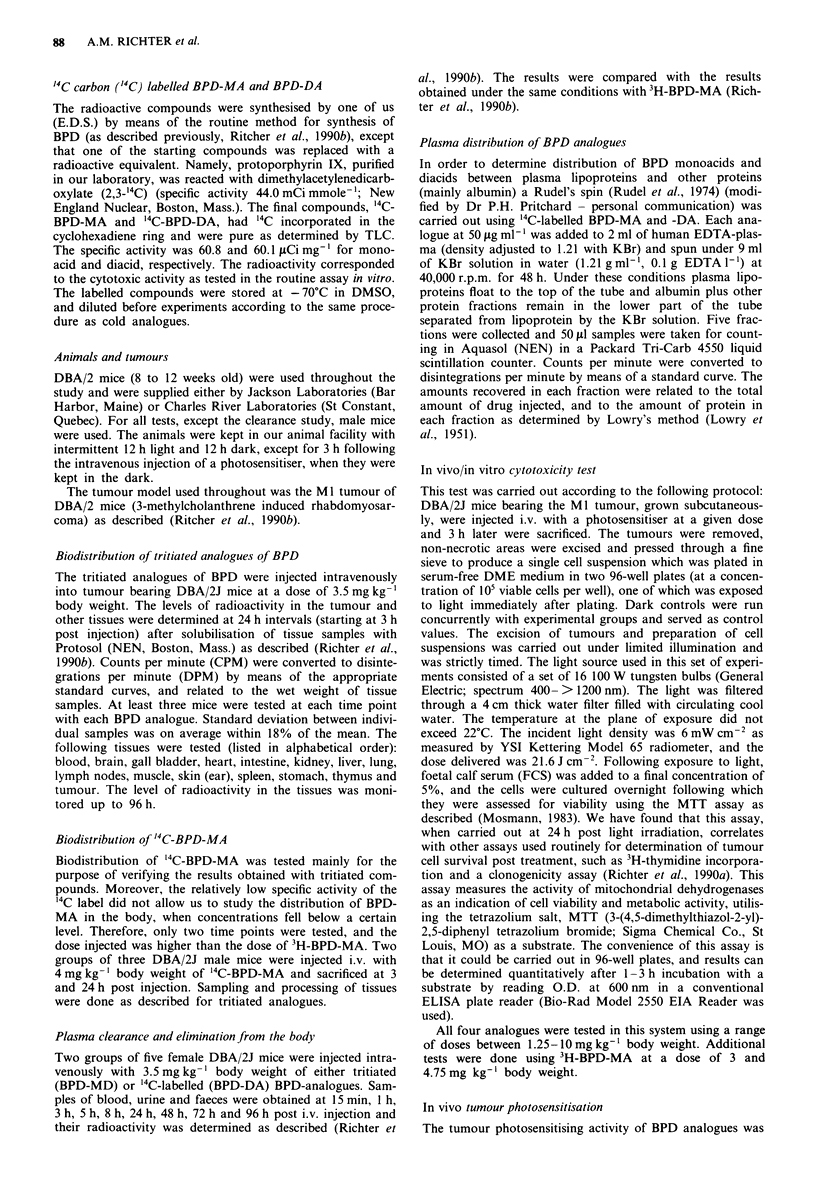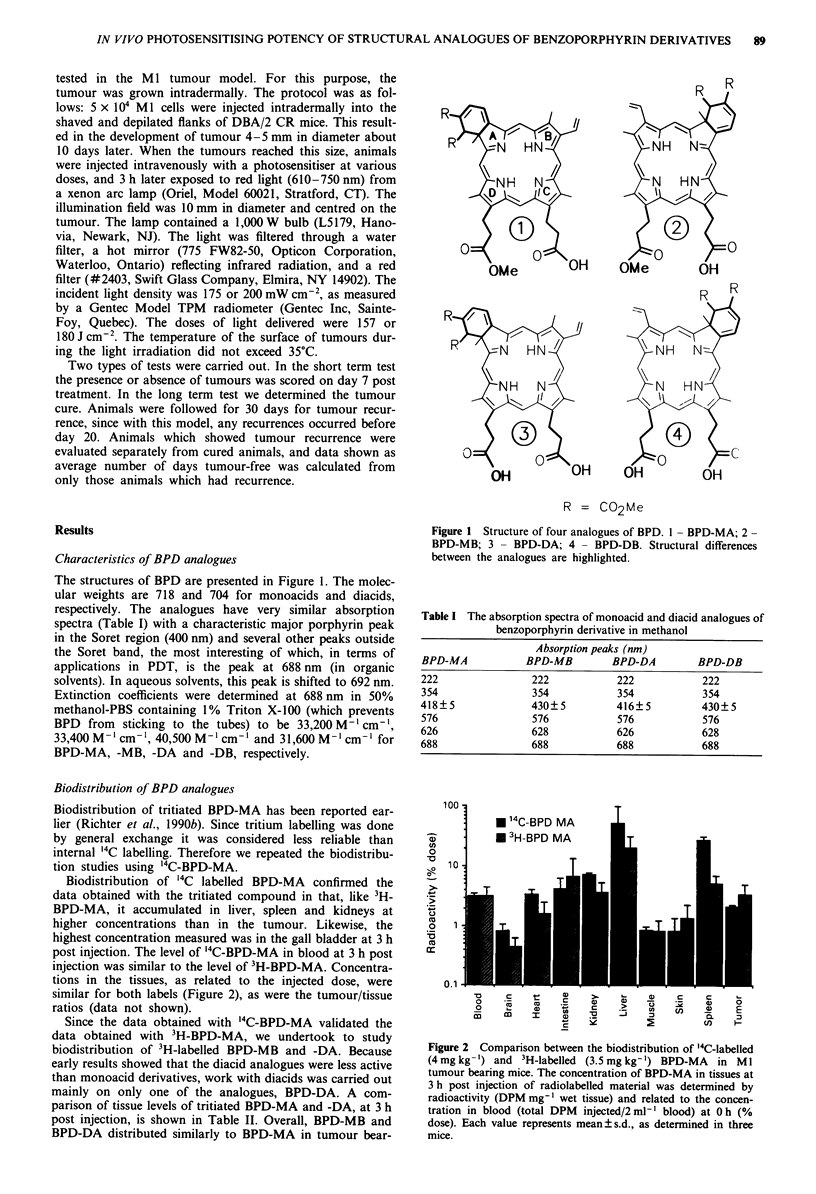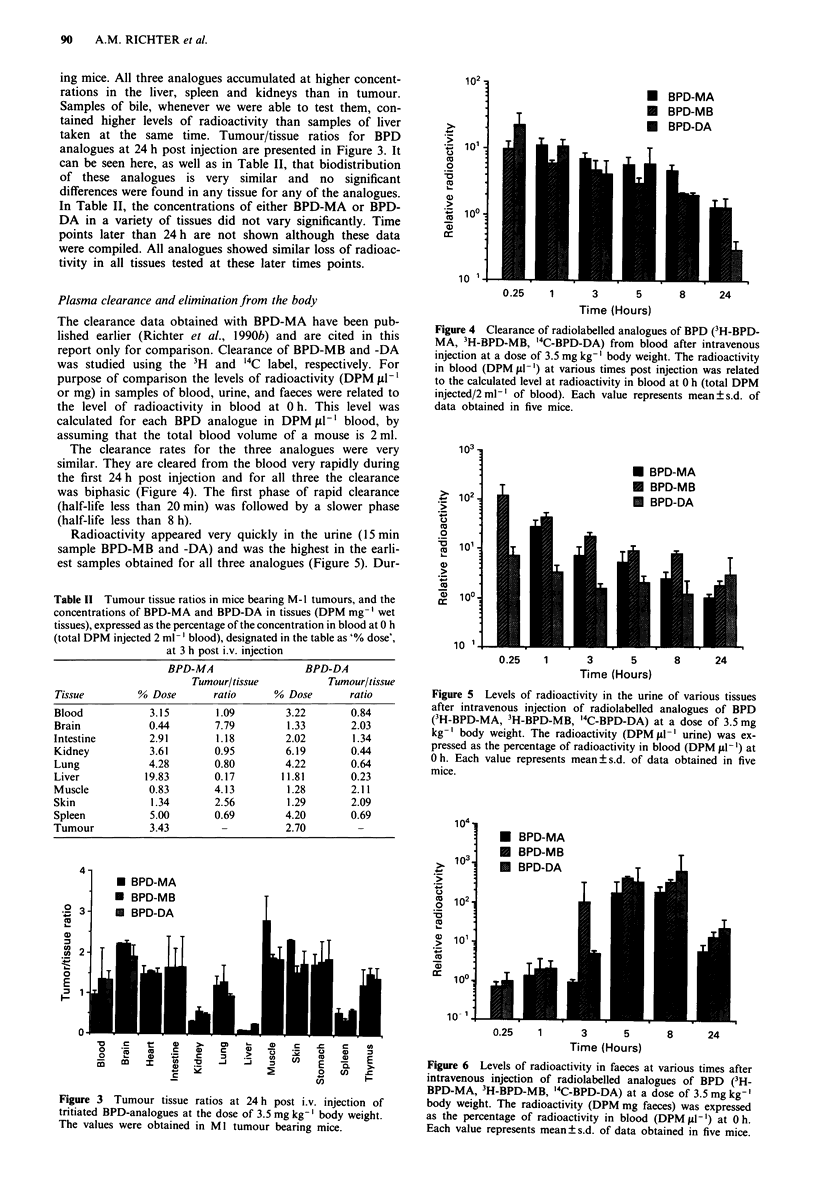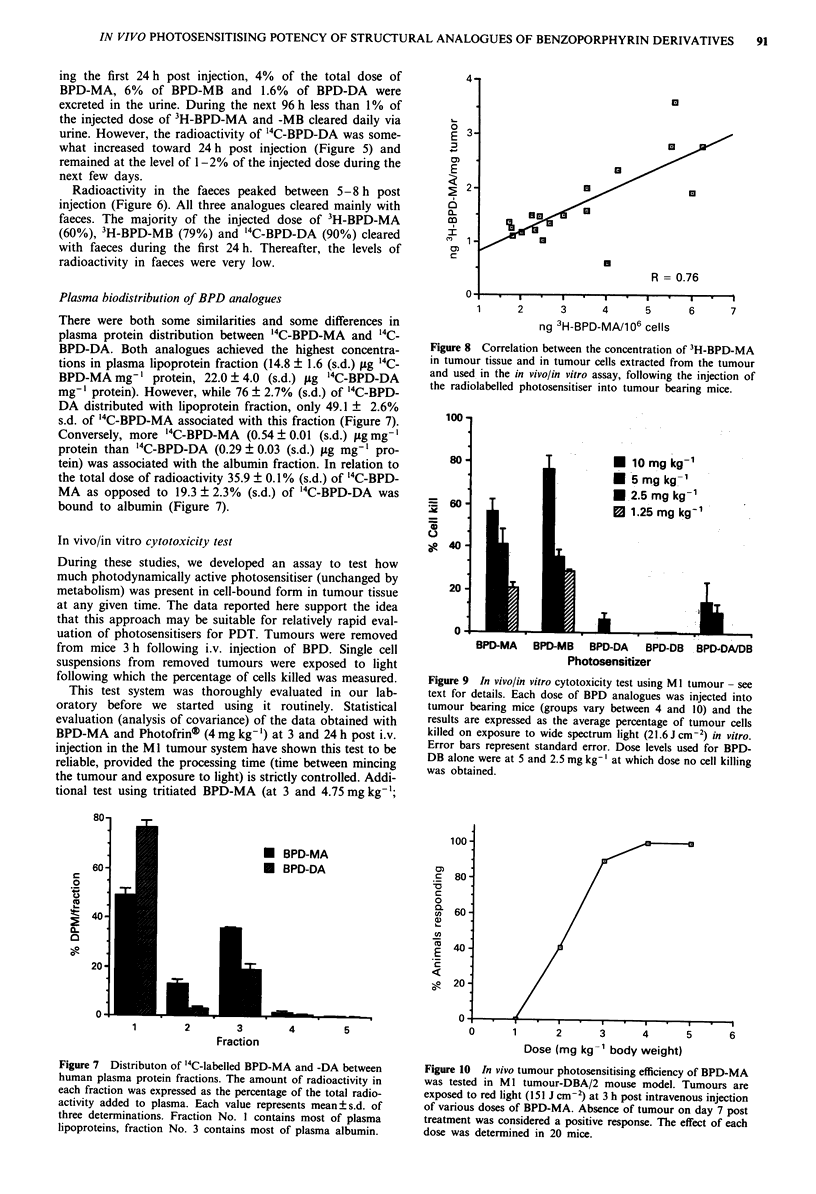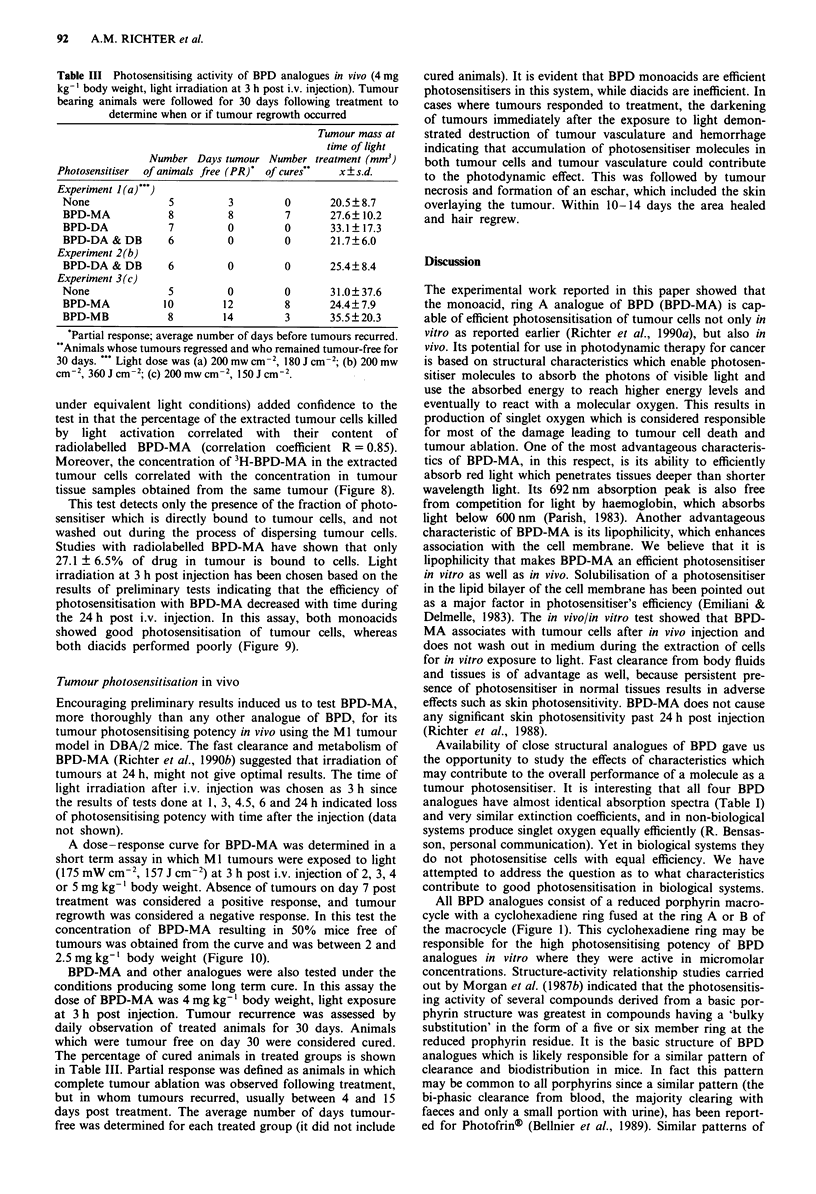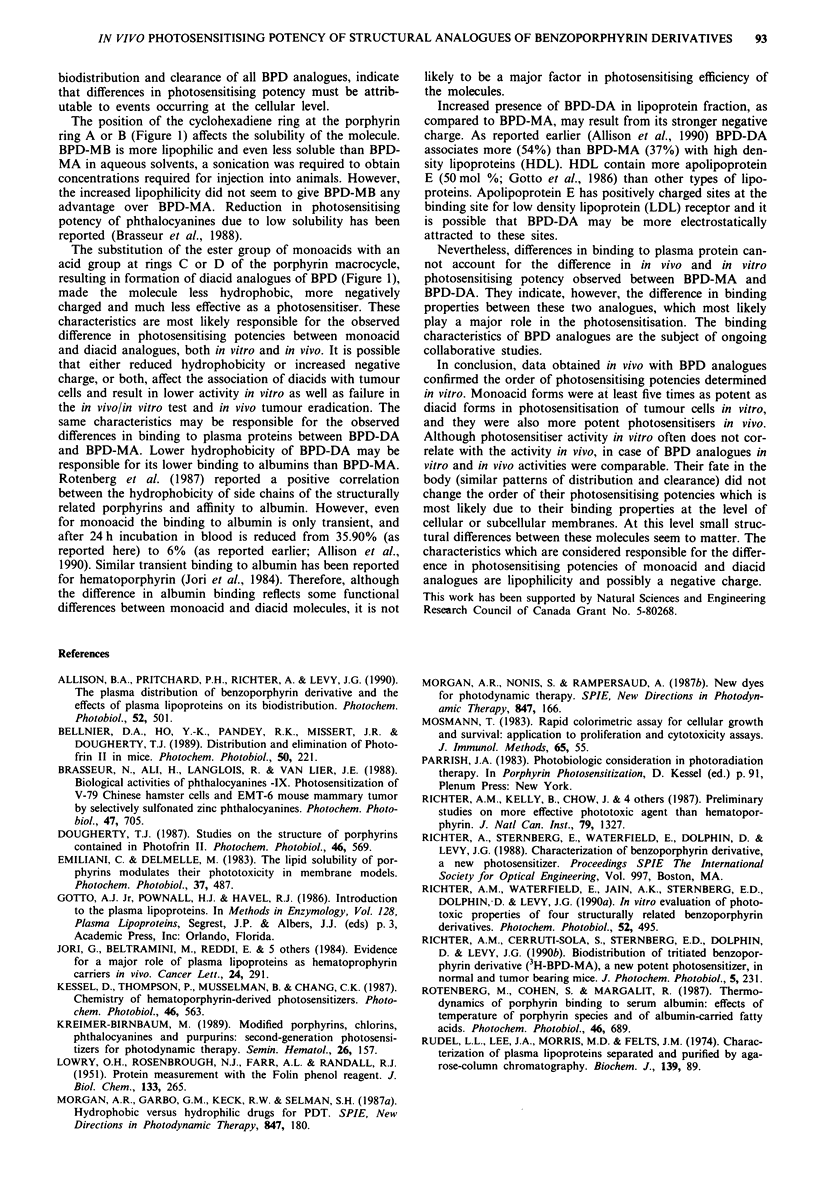# Photosensitising potency of structural analogues of benzoporphyrin derivative (BPD) in a mouse tumour model.

**DOI:** 10.1038/bjc.1991.18

**Published:** 1991-01

**Authors:** A. M. Richter, E. Waterfield, A. K. Jain, B. Allison, E. D. Sternberg, D. Dolphin, J. G. Levy

**Affiliations:** Department of Microbiology, University of British Columbia, Vancouver, Canada.

## Abstract

The in vivo characteristics of four analogues of benzoporphyrin derivative (BPD) have been investigated. Biodistribution data obtained in DBA/2J mice with BPD-MA (monoacid ring A analogue) which had been tritiated or internally labelled with 14C showed that both labelled materials acted in an essentially identical manner during the period of study. Biodistribution and clearance studies showed that relative distribution in a variety of mouse tissues was similar for all BPD analogues. M1 tumour cells (rhabdomyosarcoma in DBA/2J mice) taken from tumours excised from animals treated 3 h earlier with BPD, and tested in vitro for photosensitivity provided evidence that significant levels of photosensitiser detected in tumour was both active and associated with tumour cells. The monoacid forms of BPD were found to be much more photodynamically active in this test than were the diacid analogues. The ability of the analogues to ablate tumours in mice by photodynamic therapy was also tested. Again, BPD-MA and BPD-MB proved to be measurably better than the diacid analogues. These findings are discussed in reference to structural and physical differences between the analogues.


Br. J. Cancer (1991), 63, 87-93 © Macmillan Press Ltd., 1991

### Photosensitising potency of structural analogues of benzoporphyrin derivative (BPD) in a mouse tumour model

A.M. Richter[1], E. Waterfield[3], A.K. Jain[1], B. Allison[1], E.D. Sternberg[2], D. Dolphin[2] & J.G. Levy[1]

*Departments of [1]Microbiology and [2]Chemistry, University of British Columbia, Vancouver, BC, V6T 1W5, Canada; and [3]Quadra Logic Technologies, Vancouver, BC, V5Z 4H5, Canada.*

**Summary** The *in vivo* characteristics of four analogues of benzoporphyrin derivative (BPD) have been investigated. Biodistribution data obtained in DBA/2J mice with BPD-MA (monoacid ring A analogue) which had been tritiated or internally labelled with $^{14}C$ showed that both labelled materials acted in an essentially identical manner during the period of study. Biodistribution and clearance studies showed that relative distribution in a variety of mouse tissues was simlar for all BPD analogues. M1 tumour cells (rhabdomyosarcoma in DBA/2J mice) taken from tumours excised from animals treated 3 h earlier with BPD, and tested *in vitro* for photosensitivity provided evidence that significant levels of photosensitiser detected in tumour was both active and associated with tumour cells. The monoacid forms of BPD were found to be much more photodynamically active in this test than were the diacid analogues. The ability of the analogues to ablate tumours in mice by photodynamic therapy was also tested. Again, BPD-MA and BPD-MB proved to be measurably better than the diacid analogues. These findings are discussed in reference to structural and physical differences between the analogues.

Photodynamic therapy (PDT) is based on the observation that many photosensitisers accumulate somewhat selectively in tumour tissue where they can be activated by light at a desired wavelength. Most of the clinical work on PDT has been carried out with hematoporphyrin derivative (HPD) or Photofrin® (formerly Photofrin II), both preparations which contain a large number of porphyrin derivatives (Dougherty, 1987; Kessel *et al.*, 1987). The composite nature of these preparations, which are known to be effective for PDT, have made definitive research on precisely what characteristics of a photosensitiser contribute most to optimal selectivity in tumour uptake and cytotoxicity very difficult.

Results obtained with chemically defined photosensitisers such as various phthalocyanines, purpurins and chlorins, while very useful in expanding the knowledge of photosensitiser use in experimental PDT models, have yet to answer the difficult questions regarding which characteristics of a particular molecule are those which enable cell killing and selective delivery to tumours (Brasseur *et al.*, 1988; Kreimer-Birnbaum, 1989; Morgan *et al.*, 1987*a,b*). Undoubtedly, the knowledge of these characteristics could help in selecting and/or designing photosensitisers for PDT. It appears likely, however, that no photosensitiser will have all of the desirable properties since no doubt, efficacious PDT is dependent on a large number of variables.

We have been working with a chlorin-like photosensitiser, benzoporphyrin derivative (BPD), which is composed of four structural analogues following synthesis. All four analogues have an identical reduced tetrapyrrol porphyrin ring. They differ in two regards; the position of a cyclohexadiene ring which is fused at either ring A or B of the porphyrin, or in the presence of two acidic groups, or one acid and one ester group located at positions C and D of the porphyrins. *In vitro* characteristics of these analogues have been reported earlier (Richter *et al.*, 1990*a*). Monoacid analogues were found to be more efficient photosensitisers than the diacids. Of the two monoacids, monoacid ring B (BPD-MB) was found to be slightly more soluble than the ring A analogue (BPD-MA) and preliminary *in vivo* work indicated that this could be a problem in obtaining reliable data because of the possible presence of aggregates in formulated materials. Therefore our studies on *in vivo* photosensitisation with BPD have focused largely on BPD-MA. The results are reported in this paper. We also report the results of limited studies carried out with other BPD analogues, undertaken in order to identify special characteristics which make a molecule of a photosensitiser efficient in *in vivo* PDT.

### Materials and methods

### Synthesis of BPD analogues

BPD was synthesised as described earlier (Richter *et al.*, 1987). The length of hydrolysis of the dimethylester of A-ring or B-ring isomers with 25% hydrochloric acid dictates the final ratio between mono and diacids formed, longer hydrolysis leading to more complete conversion to the diacid form. The separation of the diacids from the monoacid analogues was carried out using column chromatography on silica gel as described earlier (Richter *et al.*, 1990*b*). The following analogues were obtained: BPD-monoacid, ring A (BPD-MA), and ring B (BPD-MB), and BPD diacid, ring A (BPD-DA) and ring B (BPD-DB).

All four BPD analogues were maintained in dimethyl sulfoxide (DMSO) at a concentration of 8 mg ml$^{-1}$. Immediately before injection into the animals they were diluted in phosphate buffered saline (PBS). The injected solution contained no more than 10% DMSO.

### Tritiated BPD analogues

Batches of BPD-MA, -MB and -DA were tritiated by NEN (Boston, Mass.) according to the procedure described earlier (Richter *et al.*, 1990*b*). Two batches of BPD-MA were labelled and the specific activities were 5.9 mCi mg$^{-1}$ (1st batch) and 5.46 mCi mg$^{-1}$ (2nd batch). Specific activities of BPD-MB and BPD-DA were 9.2 mCi mg$^{-1}$ and 6.57 mCi mg$^{-1}$, respectively. The labelled compounds were tested for purity and photosensitising activity before use as previously described (Richter *et al.*, 1990*b*). Tritiated compounds were not as stable as unlabelled compounds. During these studies, several aliquots were purified by column chromatography and rechecked for purity and photosensitising activity before use. In this work, purity and photosensitising activity were routinely assessed prior to experimental use of labelled materials.

Correspondence: J.G. Levy.
Received 15 June 1990; and in revised form 10 September 1990.

*$^{14}C$ carbon ($^{14}C$) labelled BPD-MA and BPD-DA*

The radioactive compounds were synthesised by one of us (E.D.S.) by means of the routine method for synthesis of BPD (as described previously, Ritcher *et al.*, 1990*b*), except that one of the starting compounds was replaced with a radioactive equivalent. Namely, protoporphyrin IX, purified in our laboratory, was reacted with dimethylacetylenedicarboxylate (2,3-$^{14}C$) (specific activity 44.0 mCi mmole$^{-1}$; New England Nuclear, Boston, Mass.). The final compounds, $^{14}C$-BPD-MA and $^{14}C$-BPD-DA, had $^{14}C$ incorporated in the cyclohexadiene ring and were pure as determined by TLC. The specific activity was 60.8 and 60.1 $\mu$Ci mg$^{-1}$ for monoacid and diacid, respectively. The radioactivity corresponded to the cytotoxic activity as tested in the routine assay *in vitro*. The labelled compounds were stored at $-70$°C in DMSO, and diluted before experiments according to the same procedure as cold analogues.

*Animals and tumours*

DBA/2 mice (8 to 12 weeks old) were used throughout the study and were supplied either by Jackson Laboratories (Bar Harbor, Maine) or Charles River Laboratories (St Constant, Quebec). For all tests, except the clearance study, male mice were used. The animals were kept in our animal facility with intermittent 12 h light and 12 h dark, except for 3 h following the intravenous injection of a photosensitiser, when they were kept in the dark.

The tumour model used throughout was the M1 tumour of DBA/2 mice (3-methylcholanthrene induced rhabdomyosarcoma) as described (Ritcher *et al.*, 1990*b*).

*Biodistribution of tritiated analogues of BPD*

The tritiated analogues of BPD were injected intravenously into tumour bearing DBA/2J mice at a dose of 3.5 mg kg$^{-1}$ body weight. The levels of radioactivity in the tumour and other tissues were determined at 24 h intervals (starting at 3 h post injection) after solubilisation of tissue samples with Protosol (NEN, Boston, Mass.) as described (Richter *et al.*, 1990*b*). Counts per minute (CPM) were converted to disintegrations per minute (DPM) by means of the appropriate standard curves, and related to the wet weight of tissue samples. At least three mice were tested at each time point with each BPD analogue. Standard deviation between individual samples was on average within 18% of the mean. The following tissues were tested (listed in alphabetical order): blood, brain, gall bladder, heart, intestine, kidney, liver, lung, lymph nodes, muscle, skin (ear), spleen, stomach, thymus and tumour. The level of radioactivity in the tissues was monitored up to 96 h.

*Biodistribution of $^{14}C$-BPD-MA*

Biodistribution of $^{14}C$-BPD-MA was tested mainly for the purpose of verifying the results obtained with tritiated compounds. Moreover, the relatively low specific activity of the $^{14}C$ label did not allow us to study the distribution of BPD-MA in the body, when concentrations fell below a certain level. Therefore, only two time points were tested, and the dose injected was higher than the dose of $^{3}H$-BPD-MA. Two groups of three DBA/2J male mice were injected i.v. with 4 mg kg$^{-1}$ body weight of $^{14}C$-BPD-MA and sacrificed at 3 and 24 h post injection. Sampling and processing of tissues were done as described for tritiated analogues.

*Plasma clearance and elimination from the body*

Two groups of five female DBA/2J mice were injected intravenously with 3.5 mg kg$^{-1}$ body weight of either tritiated (BPD-MD) or $^{14}C$-labelled (BPD-DA) BPD-analogues. Samples of blood, urine and faeces were obtained at 15 min, 1 h, 3 h, 5 h, 8 h, 24 h, 48 h, 72 h and 96 h post i.v. injection and their radioactivity was determined as described (Richter *et al.*, 1990*b*). The results were compared with the results obtained under the same conditions with $^{3}H$-BPD-MA (Richter *et al.*, 1990*b*).

*Plasma distribution of BPD analogues*

In order to determine distribution of BPD monoacids and diacids between plasma lipoproteins and other proteins (mainly albumin) a Rudel's spin (Rudel *et al.*, 1974) (modified by Dr P.H. Pritchard – personal communication) was carried out using $^{14}C$-labelled BPD-MA and -DA. Each analogue at 50 $\mu$g ml$^{-1}$ was added to 2 ml of human EDTA-plasma (density adjusted to 1.21 with KBr) and spun under 9 ml of KBr solution in water (1.21 g ml$^{-1}$, 0.1 g EDTA l$^{-1}$) at 40,000 r.p.m. for 48 h. Under these conditions plasma lipoproteins float to the top of the tube and albumin plus other protein fractions remain in the lower part of the tube separated from lipoprotein by the KBr solution. Five fractions were collected and 50 $\mu$l samples were taken for counting in Aquasol (NEN) in a Packard Tri-Carb 4550 liquid scintillation counter. Counts per minute were converted to disintegrations per minute by means of a standard curve. The amounts recovered in each fraction were related to the total amount of drug injected, and to the amount of protein in each fraction as determined by Lowry's method (Lowry *et al.*, 1951).

In vivo/in vitro *cytotoxicity test*

This test was carried out according to the following protocol: DBA/2J mice bearing the M1 tumour, grown subcutaneously, were injected i.v. with a photosensitiser at a given dose and 3 h later were sacrificed. The tumours were removed, non-necrotic areas were excised and pressed through a fine sieve to produce a single cell suspension which was plated in serum-free DME medium in two 96-well plates (at a concentration of $10^5$ viable cells per well), one of which was exposed to light immediately after plating. Dark controls were run concurrently with experimental groups and served as control values. The excision of tumours and preparation of cell suspensions was carried out under limited illumination and was strictly timed. The light source used in this set of experiments consisted of a set of 16 100 W tungsten bulbs (General Electric; spectrum 400 – > 1200 nm). The light was filtered through a 4 cm thick water filter filled with circulating cool water. The temperature at the plane of exposure did not exceed 22°C. The incident light density was 6 mW cm$^{-2}$ as measured by YSI Kettering Model 65 radiometer, and the dose delivered was 21.6 J cm$^{-2}$. Following exposure to light, foetal calf serum (FCS) was added to a final concentration of 5%, and the cells were cultured overnight following which they were assessed for viability using the MTT assay as described (Mosmann, 1983). We have found that this assay, when carried out at 24 h post light irradiation, correlates with other assays used routinely for determination of tumour cell survival post treatment, such as $^{3}H$-thymidine incorporation and a clonogenicity assay (Richter *et al.*, 1990*a*). This assay measures the activity of mitochondrial dehydrogenases as an indication of cell viability and metabolic activity, utilising the tetrazolium salt, MTT (3-(4,5-dimethylthiazol-2-yl)-2,5-diphenyl tetrazolium bromide; Sigma Chemical Co., St Louis, MO) as a substrate. The convenience of this assay is that it could be carried out in 96-well plates, and results can be determined quantitatively after 1 – 3 h incubation with a substrate by reading O.D. at 600 nm in a conventional ELISA plate reader (Bio-Rad Model 2550 EIA Reader was used).

All four analogues were tested in this system using a range of doses between 1.25 – 10 mg kg$^{-1}$ body weight. Additional tests were done using $^{3}H$-BPD-MA at a dose of 3 and 4.75 mg kg$^{-1}$ body weight.

In vivo *tumour photosensitisation*

The tumour photosensitising activity of BPD analogues was tested in the M1 tumour model. For this purpose, the tumour was grown intradermally. The protocol was as follows: $5 \times 10^4$ M1 cells were injected intradermally into the shaved and depilated flanks of DBA/2 CR mice. This resulted in the development of tumour 4–5 mm in diameter about 10 days later. When the tumours reached this size, animals were injected intravenously with a photosensitiser at various doses, and 3 h later exposed to red light (610–750 nm) from a xenon arc lamp (Oriel, Model 60021, Stratford, CT). The illumination field was 10 mm in diameter and centred on the tumour. The lamp contained a 1,000 W bulb (L5179, Hanovia, Newark, NJ). The light was filtered through a water filter, a hot mirror (775 FW82-50, Opticon Corporation, Waterloo, Ontario) reflecting infrared radiation, and a red filter (#2403, Swift Glass Company, Elmira, NY 14902). The incident light density was 175 or 200 mW $cm^{-2}$, as measured by a Gentec Model TPM radiometer (Gentec Inc, Sainte-Foy, Quebec). The doses of light delivered were 157 or 180 J $cm^{-2}$. The temperature of the surface of tumours during the light irradiation did not exceed 35°C.

Two types of tests were carried out. In the short term test the presence or absence of tumours was scored on day 7 post treatment. In the long term test we determined the tumour cure. Animals were followed for 30 days for tumour recurrence, since with this model, any recurrences occurred before day 20. Animals which showed tumour recurrence were evaluated separately from cured animals, and data shown as average number of days tumour-free was calculated from only those animals which had recurrence.

### Results

### *Characteristics of BPD analogues*

The structures of BPD are presented in Figure 1. The molecular weights are 718 and 704 for monoacids and diacids, respectively. The analogues have very similar absorption spectra (Table I) with a characteristic major porphyrin peak in the Soret region (400 nm) and several other peaks outside the Soret band, the most interesting of which, in terms of applications in PDT, is the peak at 688 nm (in organic solvents). In aqueous solvents, this peak is shifted to 692 nm. Extinction coefficients were determined at 688 nm in 50% methanol-PBS containing 1% Triton X-100 (which prevents BPD from sticking to the tubes) to be 33,200 $M^{-1} cm^{-1}$, 33,400 $M^{-1} cm^{-1}$, 40,500 $M^{-1} cm^{-1}$ and 31,600 $M^{-1} cm^{-1}$ for BPD-MA, -MB, -DA and -DB, respectively.

### *Biodistribution of BPD analogues*

Biodistribution of tritiated BPD-MA has been reported earlier (Richter *et al.*, 1990*b*). Since tritium labelling was done by general exchange it was considered less reliable than internal $^{14}C$ labelling. Therefore we repeated the biodistribution studies using $^{14}C$-BPD-MA.

Biodistribution of $^{14}C$ labelled BPD-MA confirmed the data obtained with the tritiated compound in that, like $^{3}H$-BPD-MA, it accumulated in liver, spleen and kidneys at higher concentrations than in the tumour. Likewise, the highest concentration measured was in the gall bladder at 3 h post injection. The level of $^{14}C$-BPD-MA in blood at 3 h post injection was similar to the level of $^{3}H$-BPD-MA. Concentrations in the tissues, as related to the injected dose, were similar for both labels (Figure 2), as were the tumour/tissue ratios (data not shown).

Since the data obtained with $^{14}C$-BPD-MA validated the data obtained with $^{3}H$-BPD-MA, we undertook to study biodistribution of $^{3}H$-labelled BPD-MB and -DA. Because early results showed that the diacid analogues were less active than monoacid derivatives, work with diacids was carried out mainly on only one of the analogues, BPD-DA. A comparison of tissue levels of tritiated BPD-MA and -DA, at 3 h post injection, is shown in Table II. Overall, BPD-MB and BPD-DA distributed similarly to BPD-MA in tumour bear-

**Figure 1** Structure of four analogues of BPD. 1 – BPD-MA; 2 – BPD-MB; 3 – BPD-DA; 4 – BPD-DB. Structural differences between the analogues are highlighted.

**Table I** The absorption spectra of monoacid and diacid analogues of benzoporphyrin derivative in methanol

| | *Absorption peaks (nm)* | | |
|---|---|---|---|
| *BPD-MA* | *BPD-MB* | *BPD-DA* | *BPD-DB* |
| 222 | 222 | 222 | 222 |
| 354 | 354 | 354 | 354 |
| 418±5 | 430±5 | 416±5 | 430±5 |
| 576 | 576 | 576 | 576 |
| 626 | 628 | 626 | 628 |
| 688 | 688 | 688 | 688 |

**Figure 2** Comparison between the biodistribution of $^{14}C$-labelled (4 mg $kg^{-1}$) and $^{3}H$-labelled (3.5 mg $kg^{-1}$) BPD-MA in M1 tumour bearing mice. The concentration of BPD-MA in tissues at 3 h post injection of radiolabelled material was determined by radioactivity (DPM $mg^{-1}$ wet tissue) and related to the concentration in blood (total DPM injected/2 $ml^{-1}$ blood) at 0 h (% dose). Each value represents mean ± s.d., as determined in three mice.

ing mice. All three analogues accumulated at higher concentrations in the liver, spleen and kidneys than in tumour. Samples of bile, whenever we were able to test them, contained higher levels of radioactivity than samples of liver taken at the same time. Tumour/tissue ratios for BPD analogues at 24 h post injection are presented in Figure 3. It can be seen here, as well as in Table II, that biodistribution of these analogues is very similar and no significant differences were found in any tissue for any of the analogues. In Table II, the concentrations of either BPD-MA or BPD-DA in a variety of tissues did not vary significantly. Time points later than 24 h are not shown although these data were compiled. All analogues showed similar loss of radioactivity in all tissues tested at these later times points.

*Plasma clearance and elimination from the body*

The clearance data obtained with BPD-MA have been published earlier (Richter *et al.*, 1990*b*) and are cited in this report only for comparison. Clearance of BPD-MB and -DA was studied using the $^{3}H$ and $^{14}C$ label, respectively. For purpose of comparison the levels of radioactivity (DPM $\mu l^{-1}$ or mg) in samples of blood, urine, and faeces were related to the level of radioactivity in blood at 0 h. This level was calculated for each BPD analogue in DPM $\mu l^{-1}$ blood, by assuming that the total blood volume of a mouse is 2 ml.

The clearance rates for the three analogues were very similar. They are cleared from the blood very rapidly during the first 24 h post injection and for all three the clearance was biphasic (Figure 4). The first phase of rapid clearance (half-life less than 20 min) was followed by a slower phase (half-life less than 8 h).

Radioactivity appeared very quickly in the urine (15 min sample BPD-MB and -DA) and was the highest in the earliest samples obtained for all three analogues (Figure 5). Dur-

**Table II** Tumour tissue ratios in mice bearing M-1 tumours, and the concentrations of BPD-MA and BPD-DA in tissues (DPM $mg^{-1}$ wet tissues), expressed as the percentage of the concentration in blood at 0 h (total DPM injected 2 $ml^{-1}$ blood), designated in the table as '% dose', at 3 h post i.v. injection

| | *BPD-MA* | | *BPD-DA* | |
|---|---|---|---|---|
| *Tissue* | *% Dose* | *Tumour/tissue ratio* | *% Dose* | *Tumour/tissue ratio* |
| Blood | 3.15 | 1.09 | 3.22 | 0.84 |
| Brain | 0.44 | 7.79 | 1.33 | 2.03 |
| Intestine | 2.91 | 1.18 | 2.02 | 1.34 |
| Kidney | 3.61 | 0.95 | 6.19 | 0.44 |
| Lung | 4.28 | 0.80 | 4.22 | 0.64 |
| Liver | 19.83 | 0.17 | 11.81 | 0.23 |
| Muscle | 0.83 | 4.13 | 1.28 | 2.11 |
| Skin | 1.34 | 2.56 | 1.29 | 2.09 |
| Spleen | 5.00 | 0.69 | 4.20 | 0.69 |
| Tumour | 3.43 | – | 2.70 | – |

**Figure 3** Tumour tissue ratios at 24 h post i.v. injection of tritiated BPD-analogues at the dose of 3.5 mg $kg^{-1}$ body weight. The values were obtained in M1 tumour bearing mice.

**Figure 4** Clearance of radiolabelled analogues of BPD ($^{3}H$-BPD-MA, $^{3}H$-BPD-MB, $^{14}C$-BPD-DA) from blood after intravenous injection at a dose of 3.5 mg $kg^{-1}$ body weight. The radioactivity in blood (DPM $\mu l^{-1}$) at various times post injection was related to the calculated level at radioactivity in blood at 0 h (total DPM injected/2 $ml^{-1}$ of blood). Each value represents mean ± s.d. of data obtained in five mice.

**Figure 5** Levels of radioactivity in the urine of various tissues after intravenous injection of radiolabelled analogues of BPD ($^{3}H$-BPD-MA, $^{3}H$-BPD-MB, $^{14}C$-BPD-DA) at a dose of 3.5 mg $kg^{-1}$ body weight. The radioactivity (DPM $\mu l^{-1}$ urine) was expressed as the percentage of radioactivity in blood (DPM $\mu l^{-1}$) at 0 h. Each value represents mean ± s.d. of data obtained in five mice.

**Figure 6** Levels of radioactivity in faeces at various times after intravenous injection of radiolabelled analogues of BPD ($^{3}H$-BPD-MA, $^{3}H$-BPD-MB, $^{14}C$-BPD-DA) at a dose of 3.5 mg $kg^{-1}$ body weight. The radioactivity (DPM mg faeces) was expressed as the percentage of radioactivity in blood (DPM $\mu l^{-1}$) at 0 h. Each value represents mean ± s.d. of data obtained in five mice.

ing the first 24 h post injection, 4% of the total dose of BPD-MA, 6% of BPD-MB and 1.6% of BPD-DA were excreted in the urine. During the next 96 h less than 1% of the injected dose of $^3$H-BPD-MA and -MB cleared daily via urine. However, the radioactivity of $^{14}$C-BPD-DA was somewhat increased toward 24 h post injection (Figure 5) and remained at the level of 1–2% of the injected dose during the next few days.

Radioactivity in the faeces peaked between 5–8 h post injection (Figure 6). All three analogues cleared mainly with faeces. The majority of the injected dose of $^3$H-BPD-MA (60%), $^3$H-BPD-MB (79%) and $^{14}$C-BPD-DA (90%) cleared with faeces during the first 24 h. Thereafter, the levels of radioactivity in faeces were very low.

### *Plasma biodistribution of BPD analogues*

There were both some similarities and some differences in plasma protein distribution between $^{14}$C-BPD-MA and $^{14}$C-BPD-DA. Both analogues achieved the highest concentrations in plasma lipoprotein fraction (14.8 ± 1.6 (s.d.) μg $^{14}$C-BPD-MA mg$^{-1}$ protein, 22.0 ± 4.0 (s.d.) μg $^{14}$C-BPD-DA mg$^{-1}$ protein). However, while 76 ± 2.7% (s.d.) of $^{14}$C-BPD-DA distributed with lipoprotein fraction, only 49.1 ± 2.6% s.d. of $^{14}$C-BPD-MA associated with this fraction (Figure 7). Conversely, more $^{14}$C-BPD-MA (0.54 ± 0.01 (s.d.) μg mg$^{-1}$ protein than $^{14}$C-BPD-DA (0.29 ± 0.03 (s.d.) μg mg$^{-1}$ protein) was associated with the albumin fraction. In relation to the total dose of radioactivity 35.9 ± 0.1% (s.d.) of $^{14}$C-BPD-MA as opposed to 19.3 ± 2.3% (s.d.) of $^{14}$C-BPD-DA was bound to albumin (Figure 7).

### In vivo/in vitro *cytotoxicity test*

During these studies, we developed an assay to test how much photodynamically active photosensitiser (unchanged by metabolism) was present in cell-bound form in tumour tissue at any given time. The data reported here support the idea that this approach may be suitable for relatively rapid evaluation of photosensitisers for PDT. Tumours were removed from mice 3 h following i.v. injection of BPD. Single cell suspensions from removed tumours were exposed to light following which the percentage of cells killed was measured.

This test system was thoroughly evaluated in our laboratory before we started using it routinely. Statistical evaluation (analysis of covariance) of the data obtained with BPD-MA and Photofrin® (4 mg kg$^{-1}$) at 3 and 24 h post i.v. injection in the M1 tumour system have shown this test to be reliable, provided the processing time (time between mincing the tumour and exposure to light) is strictly controlled. Additional test using tritiated BPD-MA (at 3 and 4.75 mg kg$^{-1}$;

**Figure 8** Correlation between the concentration of $^3$H-BPD-MA in tumour tissue and in tumour cells extracted from the tumour and used in the *in vivo/in vitro* assay, following the injection of the radiolabelled photosensitiser into tumour bearing mice.

**Figure 9** *In vivo/in vitro* cytotoxicity test using M1 tumour – see text for details. Each dose of BPD analogues was injected into tumour bearing mice (groups vary between 4 and 10) and the results are expressed as the average percentage of tumour cells killed on exposure to wide spectrum light (21.6 J cm$^{-2}$) *in vitro*. Error bars represent standard error. Dose levels used for BPD-DB alone were at 5 and 2.5 mg kg$^{-1}$ at which dose no cell killing was obtained.

**Figure 7** Distributon of $^{14}$C-labelled BPD-MA and -DA between human plasma protein fractions. The amount of radioactivity in each fraction was expressed as the percentage of the total radioactivity added to plasma. Each value represents mean ± s.d. of three determinations. Fraction No. 1 contains most of plasma lipoproteins, fraction No. 3 contains most of plasma albumin.

**Figure 10** *In vivo* tumour photosensitising efficiency of BPD-MA was tested in M1 tumour-DBA/2 mouse model. Tumours are exposed to red light (151 J cm$^{-2}$) at 3 h post intravenous injection of various doses of BPD-MA. Absence of tumour on day 7 post treatment was considered a positive response. The effect of each dose was determined in 20 mice.

**Table III** Photosensitising activity of BPD analogues *in vivo* (4 mg $kg^{-1}$ body weight, light irradiation at 3 h post i.v. injection). Tumour bearing animals were followed for 30 days following treatment to determine when or if tumour regrowth occurred

| *Photosensitiser* | *Number of animals* | *Days tumour free (PR)** | *Number of cures*** | *Tumour mass at time of light treatment (mm³) x±s.d.* |
|---|---|---|---|---|
| *Experiment 1(a)****) | | | | |
| None | 5 | 3 | 0 | 20.5±8.7 |
| BPD-MA | 8 | 8 | 7 | 27.6±10.2 |
| BPD-DA | 7 | 0 | 0 | 33.1±17.3 |
| BPD-DA & DB | 6 | 0 | 0 | 21.7±6.0 |
| *Experiment 2(b)* | | | | |
| BPD-DA & DB | 6 | 0 | 0 | 25.4±8.4 |
| *Experiment 3(c)* | | | | |
| None | 5 | 0 | 0 | 31.0±37.6 |
| BPD-MA | 10 | 12 | 8 | 24.4±7.9 |
| BPD-MB | 8 | 14 | 3 | 35.5±20.3 |

*Partial response; average number of days before tumours recurred. **Animals whose tumours regressed and who remained tumour-free for 30 days. *** Light dose was (a) 200 mw $cm^{-2}$, 180 J $cm^{-2}$; (b) 200 mw $cm^{-2}$, 360 J $cm^{-2}$; (c) 200 mw $cm^{-2}$, 150 J $cm^{-2}$.

under equivalent light conditions) added confidence to the test in that the percentage of the extracted tumour cells killed by light activation correlated with their content of radiolabelled BPD-MA (correlation coefficient R = 0.85). Moreover, the concentration of $^3$H-BPD-MA in the extracted tumour cells correlated with the concentration in tumour tissue samples obtained from the same tumour (Figure 8).

This test detects only the presence of the fraction of photosensitiser which is directly bound to tumour cells, and not washed out during the process of dispersing tumour cells. Studies with radiolabelled BPD-MA have shown that only 27.1 ± 6.5% of drug in tumour is bound to cells. Light irradiation at 3 h post injection has been chosen based on the results of preliminary tests indicating that the efficiency of photosensitisation with BPD-MA decreased with time during the 24 h post i.v. injection. In this assay, both monoacids showed good photosensitisation of tumour cells, whereas both diacids performed poorly (Figure 9).

### *Tumour photosensitisation* in vivo

Encouraging preliminary results induced us to test BPD-MA, more thoroughly than any other analogue of BPD, for its tumour photosensitising potency *in vivo* using the M1 tumour model in DBA/2 mice. The fast clearance and metabolism of BPD-MA (Richter *et al.*, 1990*b*) suggested that irradiation of tumours at 24 h, might not give optimal results. The time of light irradiation after i.v. injection was chosen as 3 h since the results of tests done at 1, 3, 4.5, 6 and 24 h indicated loss of photosensitising potency with time after the injection (data not shown).

A dose-response curve for BPD-MA was determined in a short term assay in which M1 tumours were exposed to light (175 mW $cm^{-2}$, 157 J $cm^{-2}$) at 3 h post i.v. injection of 2, 3, 4 or 5 mg $kg^{-1}$ body weight. Absence of tumours on day 7 post treatment was considered a positive response, and tumour regrowth was considered a negative response. In this test the concentration of BPD-MA resulting in 50% mice free of tumours was obtained from the curve and was between 2 and 2.5 mg $kg^{-1}$ body weight (Figure 10).

BPD-MA and other analogues were also tested under the conditions producing some long term cure. In this assay the dose of BPD-MA was 4 mg $kg^{-1}$ body weight, light exposure at 3 h post injection. Tumour recurrence was assessed by daily observation of treated animals for 30 days. Animals which were tumour free on day 30 were considered cured. The percentage of cured animals in treated groups is shown in Table III. Partial response was defined as animals in which complete tumour ablation was observed following treatment, but in whom tumours recurred, usually between 4 and 15 days post treatment. The average number of days tumour-free was determined for each treated group (it did not include cured animals). It is evident that BPD monoacids are efficient photosensitisers in this system, while diacids are inefficient. In cases where tumours responded to treatment, the darkening of tumours immediately after the exposure to light demonstrated destruction of tumour vasculature and hemorrhage indicating that accumulation of photosensitiser molecules in both tumour cells and tumour vasculature could contribute to the photodynamic effect. This was followed by tumour necrosis and formation of an eschar, which included the skin overlaying the tumour. Within 10-14 days the area healed and hair regrew.

### Discussion

The experimental work reported in this paper showed that the monoacid, ring A analogue of BPD (BPD-MA) is capable of efficient photosensitisation of tumour cells not only *in vitro* as reported earlier (Richter *et al.*, 1990*a*), but also *in vivo*. Its potential for use in photodynamic therapy for cancer is based on structural characteristics which enable photosensitiser molecules to absorb the photons of visible light and use the absorbed energy to reach higher energy levels and eventually to react with a molecular oxygen. This results in production of singlet oxygen which is considered responsible for most of the damage leading to tumour cell death and tumour ablation. One of the most advantageous characteristics of BPD-MA, in this respect, is its ability to efficiently absorb red light which penetrates tissues deeper than shorter wavelength light. Its 692 nm absorption peak is also free from competition for light by haemoglobin, which absorbs light below 600 nm (Parish, 1983). Another advantageous characteristic of BPD-MA is its lipophilicity, which enhances association with the cell membrane. We believe that it is lipophilicity that makes BPD-MA an efficient photosensitiser *in vitro* as well as *in vivo*. Solubilisation of a photosensitiser in the lipid bilayer of the cell membrane has been pointed out as a major factor in photosensitiser's efficiency (Emiliani & Delmelle, 1983). The *in vivo/in vitro* test showed that BPD-MA associates with tumour cells after *in vivo* injection and does not wash out in medium during the extraction of cells for *in vitro* exposure to light. Fast clearance from body fluids and tissues is of advantage as well, because persistent presence of photosensitiser in normal tissues results in adverse effects such as skin photosensitivity. BPD-MA does not cause any significant skin photosensitivity past 24 h post injection (Richter *et al.*, 1988).

Availability of close structural analogues of BPD gave us the opportunity to study the effects of characteristics which may contribute to the overall performance of a molecule as a tumour photosensitiser. It is interesting that all four BPD analogues have almost identical absorption spectra (Table I) and very similar extinction coefficients, and in non-biological systems produce singlet oxygen equally efficiently (R. Bensasson, personal communication). Yet in biological systems they do not photosensitise cells with equal efficiency. We have attempted to address the question as to what characteristics contribute to good photosensitisation in biological systems.

All BPD analogues consist of a reduced porphyrin macrocycle with a cyclohexadiene ring fused at the ring A or B of the macrocycle (Figure 1). This cyclohexadiene ring may be responsible for the high photosensitising potency of BPD analogues *in vitro* where they were active in micromolar concentrations. Structure-activity relationship studies carried out by Morgan *et al.* (1987*b*) indicated that the photosensitising activity of several compounds derived from a basic porphyrin structure was greatest in compounds having a 'bulky substitution' in the form of a five or six member ring at the reduced prophyrin residue. It is the basic structure of BPD analogues which is likely responsible for a similar pattern of clearance and biodistribution in mice. In fact this pattern may be common to all porphyrins since a similar pattern (the bi-phasic clearance from blood, the majority clearing with faeces and only a small portion with urine), has been reported for Photofrin® (Bellnier *et al.*, 1989). Similar patterns of biodistribution and clearance of all BPD analogues, indicate that differences in photosensitising potency must be attributable to events occurring at the cellular level.

The position of the cyclohexadiene ring at the porphyrin ring A or B (Figure 1) affects the solubility of the molecule. BPD-MB is more lipophilic and even less soluble than BPD-MA in aqueous solvents, a sonication was required to obtain concentrations required for injection into animals. However, the increased lipophilicity did not seem to give BPD-MB any advantage over BPD-MA. Reduction in photosensitising potency of phthalocyanines due to low solubility has been reported (Brasseur *et al.*, 1988).

The substitution of the ester group of monoacids with an acid group at rings C or D of the porphyrin macrocycle, resulting in formation of diacid analogues of BPD (Figure 1), made the molecule less hydrophobic, more negatively charged and much less effective as a photosensitiser. These characteristics are most likely responsible for the observed difference in photosensitising potencies between monoacid and diacid analogues, both *in vitro* and *in vivo*. It is possible that either reduced hydrophobicity or increased negative charge, or both, affect the association of diacids with tumour cells and result in lower activity *in vitro* as well as failure in the *in vivo/in vitro* test and *in vivo* tumour eradication. The same characteristics may be responsible for the observed differences in binding to plasma proteins between BPD-DA and BPD-MA. Lower hydrophobicity of BPD-DA may be responsible for its lower binding to albumins than BPD-MA. Rotenberg *et al.* (1987) reported a positive correlation between the hydrophobicity of side chains of the structurally related porphyrins and affinity to albumin. However, even for monoacid the binding to albumin is only transient, and after 24 h incubation in blood is reduced from 35.90% (as reported here) to 6% (as reported earlier; Allison *et al.*, 1990). Similar transient binding to albumin has been reported for hematoporphyrin (Jori *et al.*, 1984). Therefore, although the difference in albumin binding reflects some functional differences between monoacid and diacid molecules, it is not likely to be a major factor in photosensitising efficiency of the molecules.

Increased presence of BPD-DA in lipoprotein fraction, as compared to BPD-MA, may result from its stronger negative charge. As reported earlier (Allison *et al.*, 1990) BPD-DA associates more (54%) than BPD-MA (37%) with high density lipoproteins (HDL). HDL contain more apolipoprotein E (50 mol %; Gotto *et al.*, 1986) than other types of lipoproteins. Apolipoprotein E has positively charged sites at the binding site for low density lipoprotein (LDL) receptor and it is possible that BPD-DA may be more electrostatically attracted to these sites.

Nevertheless, differences in binding to plasma protein cannot account for the difference in *in vivo* and *in vitro* photosensitising potency observed between BPD-MA and BPD-DA. They indicate, however, the difference in binding properties between these two analogues, which most likely play a major role in the photosensitisation. The binding characteristics of BPD analogues are the subject of ongoing collaborative studies.

In conclusion, data obtained *in vivo* with BPD analogues confirmed the order of photosensitising potencies determined *in vitro*. Monoacid forms were at least five times as potent as diacid forms in photosensitisation of tumour cells *in vitro*, and they were also more potent photosensitisers *in vivo*. Although photosensitiser activity *in vitro* often does not correlate with the activity *in vivo*, in case of BPD analogues *in vitro* and *in vivo* activities were comparable. Their fate in the body (similar patterns of distribution and clearance) did not change the order of their photosensitising potencies which is most likely due to their binding properties at the level of cellular or subcellular membranes. At this level small structural differences between these molecules seem to matter. The characteristics which are considered responsible for the difference in photosensitising potencies of monoacid and diacid analogues are lipophilicity and possibly a negative charge.

This work has been supported by Natural Sciences and Engineering Research Council of Canada Grant No. 5-80268.

### References

ALLISON, B.A., PRITCHARD, P.H., RICHTER, A. & LEVY, J.G. (1990). The plasma distribution of benzoporphyrin derivative and the effects of plasma lipoproteins on its biodistribution. *Photochem. Photobiol.*, **52**, 501.

BELLNIER, D.A., HO, Y.-K., PANDEY, R.K., MISSERT, J.R. & DOUGHERTY, T.J. (1989). Distribution and elimination of Photofrin II in mice. *Photochem. Photobiol.*, **50**, 221.

BRASSEUR, N., ALI, H., LANGLOIS, R. & VAN LIER, J.E. (1988). Biological activities of phthalocyanines -IX. Photosensitization of V-79 Chinese hamster cells and EMT-6 mouse mammary tumor by selectively sulfonated zinc phthalocyanines. *Photochem. Photobiol.*, **47**, 705.

DOUGHERTY, T.J. (1987). Studies on the structure of porphyrins contained in Photofrin II. *Photochem. Photobiol.*, **46**, 569.

EMILIANI, C. & DELMELLE, M. (1983). The lipid solubility of porphyrins modulates their phototoxicity in membrane models. *Photochem. Photobiol.*, **37**, 487.

GOTTO, A.J. Jr, POWNALL, H.J. & HAVEL, R.J. (1986). Introduction to the plasma lipoproteins. In *Methods in Enzymology, Vol. 128, Plasma Lipoproteins*, Segrest, J.P. & Albers, J.J. (eds) p. 3, Academic Press, Inc: Orlando, Florida.

JORI, G., BELTRAMINI, M., REDDI, E. & 5 others (1984). Evidence for a major role of plasma lipoproteins as hematoprophyrin carriers *in vivo*. *Cancer Lett.*, **24**, 291.

KESSEL, D., THOMPSON, P., MUSSELMAN, B. & CHANG, C.K. (1987). Chemistry of hematoporphyrin-derived photosensitizers. *Photochem. Photobiol.*, **46**, 563.

KREIMER-BIRNBAUM, M. (1989). Modified porphyrins, chlorins, phthalocyanines and purpurins: second-generation photosensitizers for photodynamic therapy. *Semin. Hematol.*, **26**, 157.

LOWRY, O.H., ROSENBROUGH, N.J., FARR, A.L. & RANDALL, R.J. (1951). Protein measurement with the Folin phenol reagent. *J. Biol. Chem.*, **133**, 265.

MORGAN, A.R., GARBO, G.M., KECK, R.W. & SELMAN, S.H. (1987*a*). Hydrophobic versus hydrophilic drugs for PDT. *SPIE, New Directions in Photodynamic Therapy*, **847**, 180.

MORGAN, A.R., NONIS, S. & RAMPERSAUD, A. (1987*b*). New dyes for photodynamic therapy. *SPIE, New Directions in Photodynamic Therapy*, **847**, 166.

MOSMANN, T. (1983). Rapid colorimetric assay for cellular growth and survival: application to proliferation and cytotoxicity assays. *J. Immunol. Methods*, **65**, 55.

PARRISH, J.A. (1983). Photobiologic consideration in photoradiation therapy. In *Porphyrin Photosensitization*, D. Kessel (ed.) p. 91, Plenum Press: New York.

RICHTER, A.M., KELLY, B., CHOW, J. & 4 others (1987). Preliminary studies on more effective phototoxic agent than hematoporphyrin. *J. Natl Can. Inst.*, **79**, 1327.

RICHTER, A., STERNBERG, E., WATERFIELD, E., DOLPHIN, D. & LEVY, J.G. (1988). Characterization of benzoporphyrin derivative, a new photosensitizer. *Proceedings SPIE The International Society for Optical Engineering*, Vol. 997, Boston, MA.

RICHTER, A.M., WATERFIELD, E., JAIN, A.K., STERNBERG, E.D., DOLPHIN, D. & LEVY, J.G. (1990*a*). *In vitro* evaluation of phototoxic properties of four structurally related benzoporphyrin derivatives. *Photochem. Photobiol.*, **52**, 495.

RICHTER, A.M., CERRUTI-SOLA, S., STERNBERG, E.D., DOLPHIN, D. & LEVY, J.G. (1990*b*). Biodistribution of tritiated benzoporphyrin derivative ($^3$H-BPD-MA), a new potent photosensitizer, in normal and tumor bearing mice. *J. Photochem. Photobiol.*, **5**, 231.

ROTENBERG, M., COHEN, S. & MARGALIT, R. (1987). Thermodynamics of porphyrin binding to serum albumin: effects of temperature of porphyrin species and of albumin-carried fatty acids. *Photochem. Photobiol.*, **46**, 689.

RUDEL, L.L., LEE, J.A., MORRIS, M.D. & FELTS, J.M. (1974). Characterization of plasma lipoproteins separated and purified by agarose-column chromatography. *Biochem. J.*, **139**, 89.